# Development of a Tuneable NDIR Optical Electronic Nose

**DOI:** 10.3390/s20236875

**Published:** 2020-12-01

**Authors:** Siavash Esfahani, Akira Tiele, Samuel O. Agbroko, James A. Covington

**Affiliations:** School of Engineering, University of Warwick, Coventry CV4 7AL, UK; Siavash.Esfahani@warwick.ac.uk (S.E.); F-A.Tiele@warwick.ac.uk (A.T.); Sam.Agbroko@warwick.ac.uk (S.O.A.)

**Keywords:** electronic nose, tuneable optical sensor, non-dispersive infrared, odour fingerprints, chemical analysis, gas rig testing

## Abstract

Electronic nose (E-nose) technology provides an easy and inexpensive way to analyse chemical samples. In recent years, there has been increasing demand for E-noses in applications such as food safety, environmental monitoring and medical diagnostics. Currently, the majority of E-noses utilise an array of metal oxide (MOX) or conducting polymer (CP) gas sensors. However, these sensing technologies can suffer from sensor drift, poor repeatability and temperature and humidity effects. Optical gas sensors have the potential to overcome these issues. This paper reports on the development of an optical non-dispersive infrared (NDIR) E-nose, which consists of an array of four tuneable detectors, able to scan a range of wavelengths (3.1–10.5 μm). The functionality of the device was demonstrated in a series of experiments, involving gas rig tests for individual chemicals (CO_2_ and CH_4_), at different concentrations, and discriminating between chemical standards and complex mixtures. The optical gas sensor responses were shown to be linear to polynomial for different concentrations of CO_2_ and CH_4_. Good discrimination was achieved between sample groups. Optical E-nose technology therefore demonstrates significant potential as a portable and low-cost solution for a number of E-nose applications.

## 1. Introduction

The electronic nose (E-nose) has been in continuous development since its conception in the early 1980s by Persaud and Dodd [1]. The term E-nose describes an instrument consisting of an array of cross-sensitive gas sensors, coupled with a pattern recognition approach. This operating principle attempts to mimic the function of biological olfactory receptors by analysing samples as a whole (so-called “odour fingerprints” [2]), rather than individual chemicals. In the past, E-noses were mainly used in the food safety or food quality, for example, for the determination of tea quality, adulteration of olive oil, fruit ripening and the rancidity of meat [3]. More recently, two other major application areas for E-noses have emerged; specifically, environmental monitoring (e.g., detecting pollutants, hazardous chemicals and/or explosives) [4] and biomedical purposes (e.g., monitoring and diagnosing diseases) [5]. These applications rely on the ability of E-noses to generate a holistic analysis of a gas-phase mixture, which are made up of volatile organic compounds (VOCs) and some non-VOCs, such as inorganic gases. VOCs can be broadly defined as carbon-based compounds (C_2_–C_30_), which include a diverse group of compounds such as hydrocarbons, esters, alcohols, ketones and aldehydes, with high vapour pressures and low boiling points (50–260 °C) [6].

In gas-phase chemical sensing, the gold standard is generally considered to be gas chromatography mass spectrometry (GC-MS) [7]. While this method is highly reproducible and accurate, it is also very expensive, requires highly trained staff and lacks portability. The key advantage of E-noses is that their relatively simple operating principle and construction enables far more flexibility in the tailoring of the design and technical specification (e.g. ease of use, battery life, durability, number of sensors, sensing technology and sampling technique). There are several types of sensor technologies suitable for constructing E-nose arrays. An in-depth summary can be found in the review by Dospinescu et al. [8]. The most commonly used sensors in E-noses are metal oxide (MOX) and conducting polymers (CP). Both types of sensors operate through a change in conductivity when exposed to a target gas [9,10]. Examples of commercial E-noses, based on MOX and CP technology, include PEN3 [11] (AIRSENSE Analytics, Schwerin, Germany), FOX4000 [12] (AlphaMOS, Toulouse, France) and Cyranose 320 [13] (Sensigent, Baldwin Park, CA, USA). While these E-noses have been successfully deployed in a number of studies [14], a limitation is that they can suffer from drift and poor repeatability. MOX sensor responses can drift significantly, due to ambient temperature, pressure changes and material aging [15], and CP sensors often demonstrate poor repeatability and reproducibility, due to the random nature of the polymer [16]. The effects of sensor drift can be mitigated by utilising multi-sensor arrays (e.g., 6–18 sensors), to reduce the relative drift of individual sensors within the array, and by using pattern recognition techniques to account for material drift and environmental changes. Repeatability issues can be addressed by calibrating the E-nose to a number of standardised gas exposures. While these methods are relatively well-established, other sensing technologies are available that are fundamentally less susceptible to these effects, such as optical gas sensors.

Optical gas sensors measure the modulation of light properties or characteristics, such as changes in absorbance, polarisation, fluorescence or other optical properties [17]. Since this method does not relate to chemical reactions, such as those for MOX and CP sensors, environmental changes have less effect on the response of optical sensors, with the exception of pressure. In general, optical gas sensors therefore require less frequent calibration. Moreover, with regard to sensor drift, single, or much smaller numbers of optical gas sensors, could perform similarly to larger MOX or CP sensor arrays and would also be less reliant on pattern recognition techniques to account for environmental changes. Further advantages of this sensing mechanism are that it is highly sensitive to a wide range of VOCs, often have good selectivity (especially to gases such as CO_2_ and CH_4_), good response/recovery times and longer sensor life than other gas sensor types [18,19]. The disadvantages of optical sensors are that they are typically more expensive and can have lower portability, due to the delicate optics and electronics [4]. Moreover, they are difficult to miniaturise and may require complex electrical circuits, since they do not rely on measuring a simple transduction mechanism, such as electrical resistance [19]. Despite some of these limitations, it has been argued that optical sensors are fundamentally among those best suited for E-nose applications [5].

Compared to the other gas sensor technologies, optical sensors have had less commercial success, likely due to cost and level of sensitivity, with the exception of targeted CO_2_ and CH_4_ detection [20]. Review papers evaluating the application of E-noses in food safety focus entirely on MOX and CP sensor-based system and merely mention optical technology in a long list of potential sensing methods [21,22]. Similar trends are observed in review articles that focus on the application of E-noses for environmental monitoring [4,23] and medical diagnostics [24,25]. These reviews indicate that E-noses are currently under-utilised in research and that there is still significant scope to evaluate the potential of this technology. In the commercial domain, there is a small selection of gas analysers, which utilise optical technology, such as the IRIS 4100 mid-IR and MIRAN SapphIRe (Thermo Fisher Scientific, Waltham, MA, USA). The former uses laser absorption spectroscopy to measure CO_2_, while the latter uses a single beam infrared (IR) spectrophotometer for the monitoring of ambient air samples. The MIRAN SapphIRe unit is portable and can detect the presence of up to 121 gases, but is also expensive (upwards of $30,000). Further examples of portable gas analysers include the GT5000 Terra, DX4015 and DX4000 (Gasmet Technologies, Vantaa, Finland). These operate based on Fourier Transform IR (FTIR) spectroscopy and can measure up to 50 gases simultaneously, but they cost upwards of $40,000. These instruments exemplify the current state of commercial optical sensors, in that the devices are too costly or specialised.

The growing demand for Internet-of-things (IoT) enabled environmental monitors [26], point-of-care diagnostic devices [24] and real-time food quality sampling equipment [27] calls for novel E-nose designs and concepts. These should enable E-noses to operate in environmentally challenging conditions, with continuous temperature and humidity fluctuations. While current MOX and CP-based gas sensors may struggle in these situations, optical sensing technology has the potential to thrive. The aim of this work is therefore to develop a novel optical-based E-nose, which is portable and can be deployed in a wide variety of typical E-noses applications.

## 2. Materials and Methods

**Figure 1:**
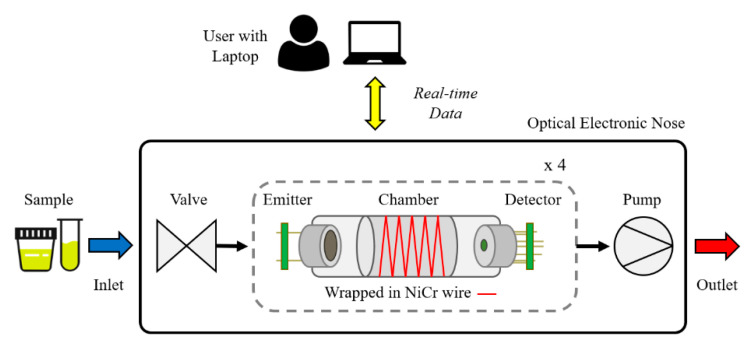
Optical electronic nose system diagram.

The proposed optical E-nose is comprised of four emitters, paired with tuneable detectors. Each emitter–detector pair is individually encapsulated in a heated sensor chamber. The sample inlet is controlled using a valve and the system operates a negative-pressure system, using a pump at the exhaust. The system is controlled by the user using a laptop, via wired USB connection. A system diagram of the optical E-nose is shown in Figure 1.

The following section discusses the individual sub-systems of the device in more detail.

### 2.1. Optical Gas Sensors

**Figure 2:**
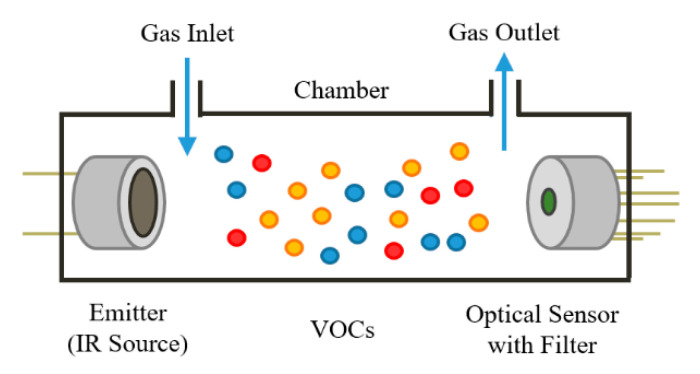
Optical gas sensing principle.

A nondispersive infrared (NDIR) sensor system consists of three main parts: emitter (IR source), gas flow path and IR detector (with filter) (Figure 2). The operating principle is based on molecular absorption spectrometry. When a gas is inside the chamber, molecules absorb the radiation from the IR source. The filter on the optical sensor will only let the radiation wavelength related to the target gas pass. Molecular absorption of IR is measured to detect a fall in signal, which can produce a unique odour fingerprint [28]. Several VOCs relevant for food safety and biomedical monitoring applications are known to have absorption frequencies in the range of 2.8–5.2 μm, such as acetone, 2-butanone and isopropanol [29,30]. It is worth noting that the number of photons absorbed is directly proportional to the power of the photon beam from the emitter and thus the amount of the gas/vapour detected. It is therefore possible to determine the concentration of the measured molecule. There are two advantages associated with IR radiation. Firstly, IR radiation scatters less than visible radiation in the presence of steam, mist or smoke, due to its longer wavelength [31]. Secondly, the miniaturisation of IR sources and detectors is possible using the latest MEMS technology.

The optical E-nose utilises commercially available emitters and detectors. The emitters are high emissivity thermal IR emitters (EMIRS200, Axetris, Kägiswil, Switzerland). The tuneable detectors were developed by InfraTec (Dresden, Germany) and are summarised in Table 1.

These detectors have the ability to scan the mid- and long-wave IR range (3.1–10.5 μm) [32], in steps of 20 nm. This should be considered the wavelength resolution of the optical E-nose. Due to this feature, any fixed frequency measurements can be considered as “virtual sensors”. Conventional IR detectors use expensive precision filters to select specific frequencies [33]. Modern IR detectors use a filtering technique based on micromachined Fabry–Pérot interferometer (FPI), which involves an optical cavity with two parallel reflecting surfaces, which act as a half-wave resonator [34]. By changing the plate separation, the central wavelength can be tuned [35]. This can be achieved by simply adjusting the voltage across a control pin. Since gases/VOCs have a unique infrared absorption frequency [36], this optical E-nose can be used to identify individual gases/VOCs in a complex sample, as well as analysing the odour as a whole without identification of each chemical [37].

### 2.2. Sensor Chamber Design and Heating

The emitter–detector pairs are encapsulated in individual, heated chambers. Gas chambers for IR sensors can be designed in two different ways, intended to either maximise resolution or signal-to-noise ratio (SNR). Since most applications of E-noses require the detection of low concentrations of VOCs (ppm to ppb range), the high-resolution design was chosen. The length of the chamber is a critical feature of this design. According to Beer–Lambert law, increasing the optical path will increase sensitivity, but this decreases the SNR from emitter to detector. Different sensor chamber lengths were evaluated to determine the optimal length of the selected sensors. Lengths under consideration were 10, 20, 30, 40 and 50 cm. The results from these experiments demonstrate that the 30 cm chamber was associated with the highest differential voltage sensor response, using CO_2_ as the test gas. The term “differential voltage sensor response” refers to the difference between the baseline (ambient air) sensor response vs. signal when exposed to an analyte—as measured in Volts (or milli-Volts). The voltage drops in the presence of 1000 ppm CO_2_ (absorption frequency set to 4.2 μm) are summarised in Table 2.

**Figure 3:**
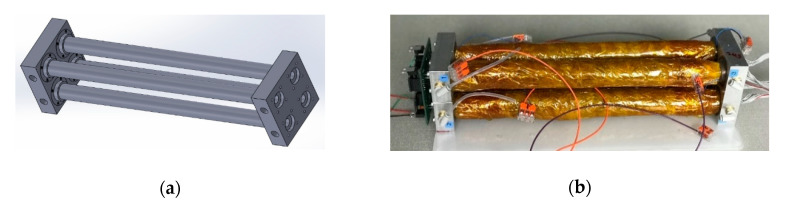
Optical electronic nose 2 × 2 gas sensor chamber formation: (**a**) 3D model; and (**b**) manufactured prototype.

The sensor chamber length was manufactured to a length of 30 cm. The four chambers were arranged in a 2 × 2 formation, with mounting plates on either end, as shown in Figure 3. An alternative approach could involve fitting all four emitters and detectors into a single wider chamber; however, this is likely to result in poor SNR.

The sensor chambers are made of stainless-steel tubes. The internal surface was polished, which results in more reflections. The material is also relatively easy to clean and has good thermal characteristics to implement an effective chamber heating system. A heating system is necessary in order to avoid condensation forming inside the chambers, thereby reducing environmental effects on the sensors. By having a fixed temperature above ambient, thermal effects of different sample temperatures is reduced. However, this also reduces the SNR.

**Figure 4:**
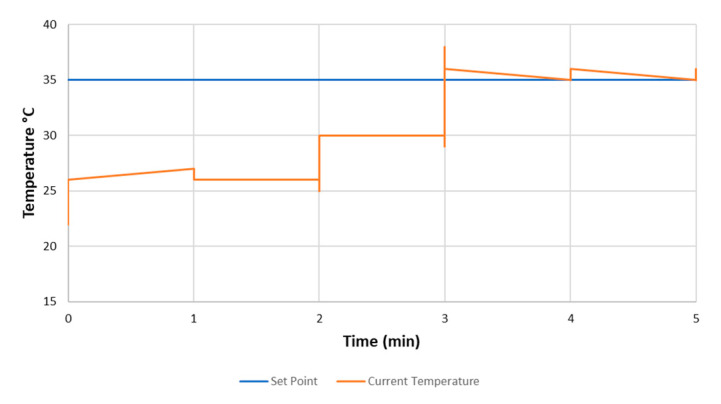
Internal chamber temperature increasing to the user-defined set-point of 35 °C.

Nichrome (NiCr) wire was used as a heating element, since it is low-cost and simple to implement and control. NiCr wire was coiled around each of the chambers and wrapped in high temperature resistant Kapton tape (436-2778, RS, Corby, UK) to provide some insulation and prevent short circuits. The NiCr wire’s heat dissipation is in direct relation to the amount of voltage and current applied to it. The temperature inside the chamber is automatically regulated using a 24 V supply and a PID controller algorithm (proportional, integral and differential). The temperature set-point is selected by the user (e.g., 35 °C) and feedback from inside the chamber is provided by a SHT75 temperature and humidity sensor (Sensirion, Stäfa, Switzerland). The maximum temperature should not exceed 65 °C so as not to damage the sensors. The heating procedure is initiated during start up and takes round 5 min, as shown in Figure 4.

### 2.3. Electronic Design and Software

This system can be controlled by a tablet or PC, via wired USB connection. A custom app was created using Universal Windows Platform (UWP) to view the sensor responses in real-time and change system properties (e.g., chamber temperature). The UWP app communicates with a central microcontroller, a Teensy 3.6 (PJRC, Portland, OR, USA) development board. This is used to interface with the emitter and detector boards (using UART), as well as the rest of the control electronics. The electronic design requirements for the optical E-nose are divided into four segments: (1) sensor drive; (2) emitter drive; (3) heater system; and (4) control electronics.

The sensor drive was designed to run all four emitter/detector pairs. The sensors have different supply voltage requirements: 3.3, ±5, and 12 V. The electronic drive board was designed to work with 24 V and regulated to lower and negative voltages using linear voltage regulators. In addition, depending on the sensor, a voltage of 30–90 V was required for controlling the central wavelength; specifically, 30 V for LFP3144C-337, 45 V for LFP-3850C-337, 60 V for LFP-8850-337 and 90 V for LFP-80105C-337. The voltage regulators used to generate 12, 5, 3 and −5 V were LM2937IMP, L78L05ABD, LD1117S33TR and L79L05ABD, respectively. To generate 90 V from 12 V, a LT1372HVCS8 high frequency switching regulator was used in combination with a flyback transformer.

**Figure 5:**
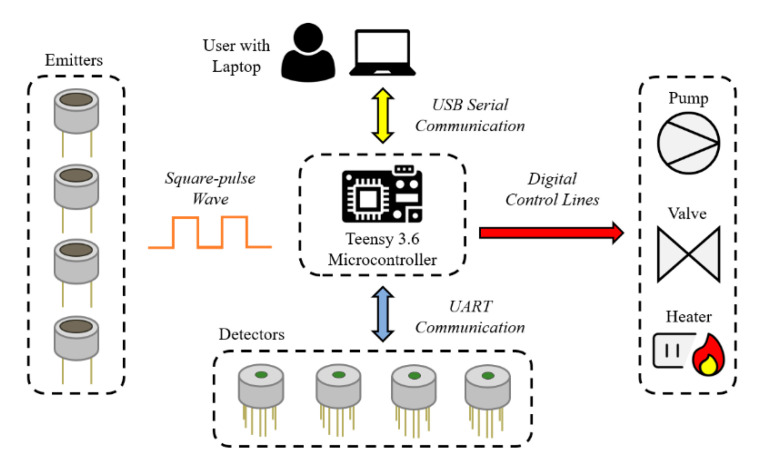
Overview of the optical electronic nose communication/control lines.

The emitter drive implemented a power-regulated circuit. This approach can compensate the inherent variation in source parameters and is more efficient for DC voltages with low frequency pulses. A square-pulse wave from the Teensy 3.6 was used to turn on and off the emitters, at a frequency of 10 Hz. The heater system regulates the temperature of the sensor chambers. A simple switching circuit turns the voltage on/off across the NiCr wire. A digital control signal from the Teensy 3.6 is used to turn on/off a MOSFET, which allows current to pass through the NiCr wire, when the temperature reading falls below a predetermined threshold. The control electronics provide 24 V to the four heated sensor chambers, sensor circuitry and 12 V to the pump and valve. Two input power adaptors were used to separate the heating input power from the control circuitry. This is because of the high current required for the heater system. An overview of the optical E-nose communication/control lines is shown in Figure 5.

### 2.4. Mechanical Design

**Figure 6:**
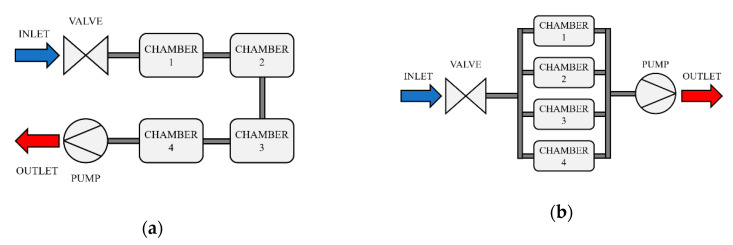
Optical electronic nose airflow pathways: (**a**) series configuration; and (**b**) parallel configuration.

Mechanical elements of the optical E-nose include the internal flow path configuration and mounting of components inside an electronics enclosure. The flow path within the optical E-nose can be configured in two ways, as shown in Figure 6. The sample flow can pass through the sensor chambers either in series or in parallel. Both versions have their advantages and disadvantages. In both variants, a valve controls the sample inlet and a pump pulls the sample through the device, to create a negative pressure system. In the series version, the pump flowrate is crucial for the sensor readings. If the sample passes through the system too quickly, the sensors may not be able to produce an accurate and reproducible reading. The advantage of this configuration is that the total amount of VOCs is present across all chambers during analysis. In the parallel configuration, the sample will be split. Since the sample is divided by the number of chambers (in this case four), dilution and marginal pressure differences may occur from chamber to chamber. The advantage of this version is that all sensors will have sufficient time to detect the VOCs in the sample. The current version of the optical eNose utilises the parallel flow path configuration.

**Figure 7:**
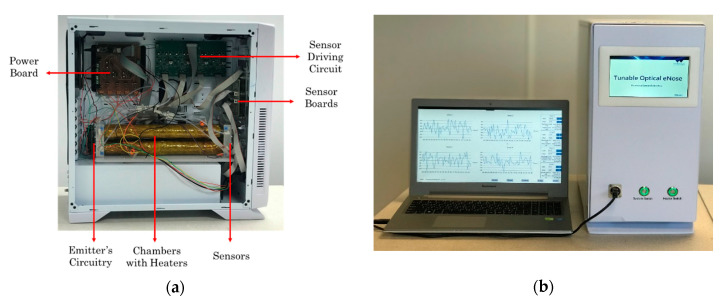
Optical electronic nose: (**a**) internal view; and (**b**) external view, with laptop.

**Figure 8:**
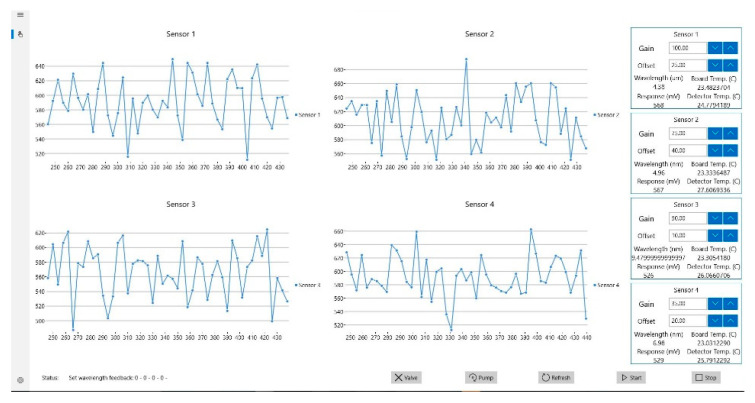
Optical electronic nose graphical user interface.

The optical E-nose was designed and constructed to match the form factor of traditional E-noses. The completed system weighs around 12 kg and the dimensions of the white electronics enclosure are 48 cm × 23 cm × 47 cm. The circuit boards and sensor chamber were mounted inside the enclosure using 3 mm acrylic sheet support structures (434-295, RS, Corby, UK). Internal and external views of the unit are shown in Figure 7. A screenshot of the graphical user interface (GUI) is shown in Figure 8.

## 3. Results

Two sets of experiments were conducted to evaluate the developed innovative optical E-nose. The first used was a gas rig test, using individual gases, whereby the concentration of the gas is altered in order to observe the sensitivity of the sensors. The second was a sample group discrimination test, whereby different simple chemical standards and complex odour mixtures were analysed. The first set of tests was intended to develop an understanding of how the optical sensors respond to different concentration changes. The second set of tests replicate the most common application of E-noses—utilising a pattern recognition approach to distinguish between different sample groups.

### 3.1. Gas Rig Testing

The optical E-nose was evaluated using a gas rig to test the response to single gases, at different concentrations. CO_2_ and CH_4_ were chosen for these experiments, since they are the most commonly targeted gases for optical sensing applications. NDIR sensors are generally used for these gases due to their relatively large molar absorption coefficients [38]. Gas cylinders with nominal concentrations of 1000 ppm CO_2_ and 100 ppm CH_4_ were used. The gases were diluted with zero-air from a zero-air generator (HPZA-7000-220, Parker, Warwick, UK). The total flow rate of the diluted gas was set to 300 mL/min. For the CO_2_ test, the concentration of CO_2_ was increased linearly from 25 to 250 ppm, in steps of 25 ppm. Additionally, concentrations of 500, 750 and 1000 ppm were tested. For the CH_4_ test, the concentration of CH_4_ was increased linearly from 2.5 to 25 ppm, in steps of 2.5 ppm. Concentrations of 50, 75 and 100 ppm were also tested. For both tests, the exposure and recovery periods were 25 min between concentration steps. CO_2_ has an absorption frequency of 4.2 μm, while CH_4_ has two absorption frequencies in the IR spectrum (3.4 and 7.8 μm). Zero-air was used during recovery for E-nose responses to return to baseline levels.

**Figure 9:**
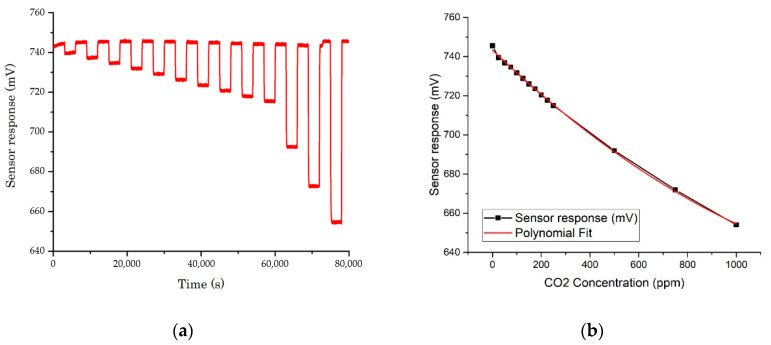
Sensor response to CO_2_ gas with different concentrations, from 25 to 1000 ppm, at 4.2 μm: (**a**) sensor response; and (**b**) polynomial fit (R-Square = 0.999).

The changes in concentration of CO_2_ vs. sensor response are shown in Figure 9. The sampling rate of the optical E-nose was set to 1 Hz (1 sample per second). Sensor responses are presented using a 100 data-point moving-average. This indicates that the sensor response is linear to polynomial (second degree) for changes in CO_2_ concentration. The plot in Figure 9 suggests that the limit of detection for CO_2_, using this technology is in the low ppm range.

**Figure 10:**
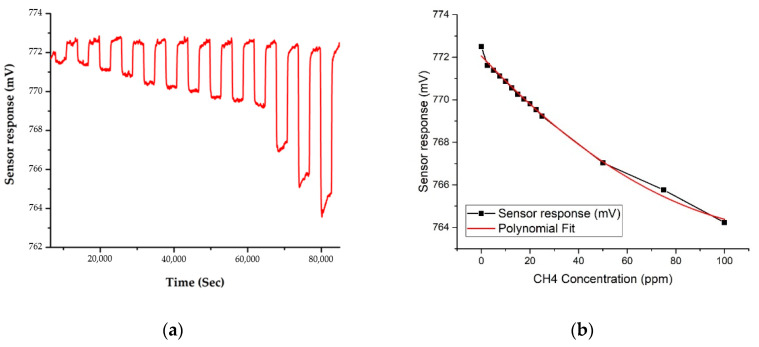
Sensor response to CH_4_ gas with different concentrations, from 2.5 ppm to 100 ppm, at 3.4 μm: (**a**) sensor response; and (**b**) polynomial fit (R-Square = 0.995).

**Figure 11:**
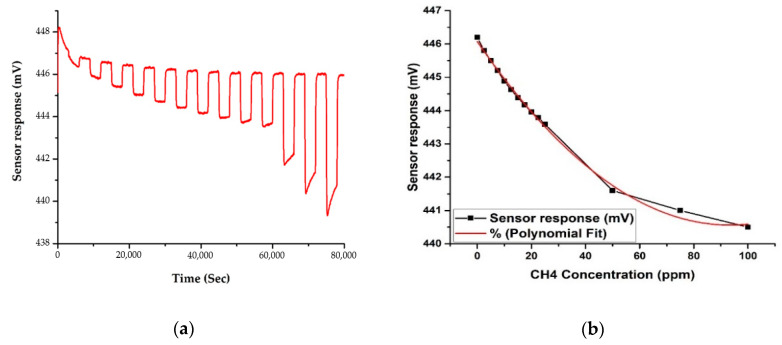
Sensor response to CH_4_ gas with different concentrations, from 2.5 ppm to 100 ppm, at 7.8 μm: (**a**) sensor response; and (**b**) polynomial fit (R-Square = 0.997).

The changes in concentration of CH_4_ vs. sensor response are shown in Figure 10 and Figure 11, for the two frequencies. These results were gathered simultaneously, thus demonstrating the system’s ability to measure multiple sensor outputs concurrently. Like CO_2_, the sensor responses show that the response is linear to polynomial (second degree) for changes in CH_4_ concentration. The plot in Figure 11 suggests that the projected sensitivity to CH_4_, as indicated by the drop in sensor response from baseline levels prior to the first CH_4_ reading, is less than 1 ppm. The limit of detection for CH_4_ using this technology must therefore also be below 1 ppm.

### 3.2. Simple and Complex Chemical Testing 

While CO_2_ and CH_4_ have value in environmental, agricultural and medical applications [39,40], there are also other compounds, such as acetone, ethanol and isopropanol, which are more relevant in the food quality and biomedical domain [21,41,42,43]. These compounds are associated with known absorption frequencies, which fall within the range of the optical E-nose system. For example, acetone and ethanol both produce four distinct absorption peaks within 3.1–10.5 μm. All four detectors could therefore simultaneously measure these VOCs, at different wavelengths.

The first set of these experiments involved a single chemical; specifically, 1 mL acetone (99% purity from Sigma-Aldrich, Dorest, UK) mixed with 4 mL of water. The 5 mL sample was aliquoted into a 20 mL glass vial and heated for 10 min at 40 ± 0.1 °C in a heater block (DB-2D Dri-Block, Techne/Cole-Parmer, Stone, UK). The sample headspace was collected using a 10 mL syringe and injected into the inlet port of the E-nose system. These concentrations were used as they are the calibration standards recommended by electronic nose manufacturers.

**Figure 12:**
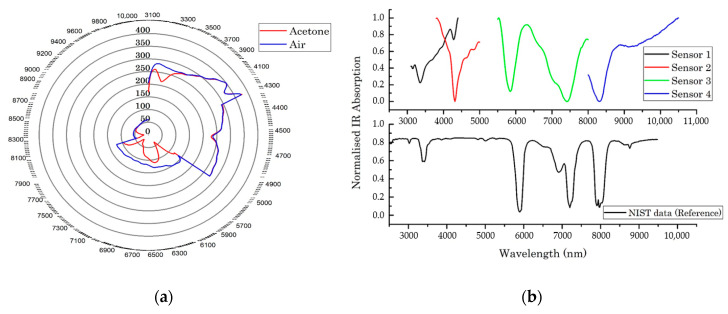
Optical electronic nose responses: (**a**) ambient air and acetone in water, across the entire infrared range (3.1–10.5 μm); and (**b**) acetone absorption frequencies.

The sensor response of diluted acetone was compared to an ambient air sample, across the entire IR range, as shown in Figure 12a. Ambient air was used as a reference sample, since this represents the baseline sensor response. This result clearly demonstrates that the response of the optical E-nose array can differentiate between the acetone and air sample. The differences are mainly observed within the absorption frequencies 3.16–3.70 and 5.56–9.00 μm.

As shown in Figure 12b, the results from the optical E-nose closely match the NIST database reference absorption profile. Since the optical E-nose has slightly different baseline values, the data from the experiments were normalised to a scale of 0–1. Sensor 1 covers the range 3.1–4.4 μm and detected acetone at 3.34 μm, which is similar to the database reference. Wavelength of Sensors 1 and 2 overlap within 3.8–4.4 μm. The absorption peak for both sensors was 4.28 μm. This is due to ambient CO_2_ gas not being removed by the zero-air generator. Sensor 3, which covers the wavelength range of 5.5–8.0 μm, shows two absorption peaks around 5.88 and 7.44 μm. This is close to absorption frequencies of the NIST database. Sensor 4, which covers the wavelength range 8.0–10.5 μm, detects acetone around 8.1 μm. In summary, the results in Figure 12b demonstrate, to a satisfactory degree, that the sensor array is able to detect acetone at all expected wavelengths.

**Figure 13:**
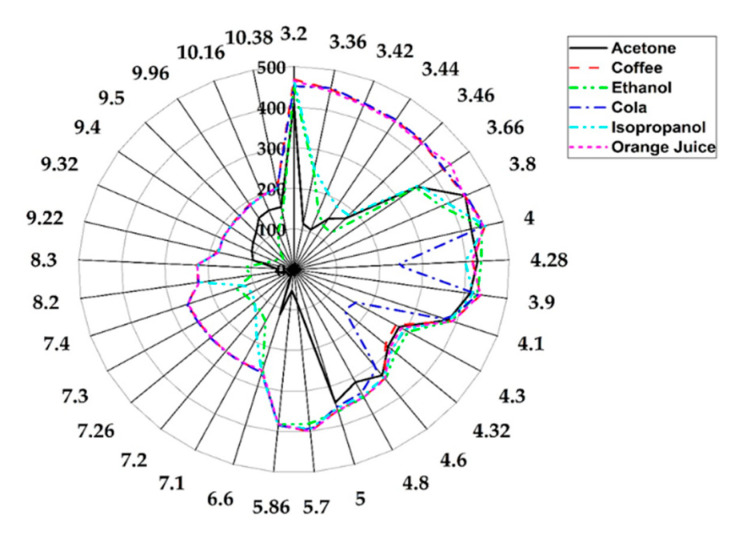
Radar plot of raw values of sensor responses for chemical standards and complex mixtures, across the entire infrared range (3.1–10.5 μm).

Following these experiments, more complex mixtures were also analysed. Figure 13 shows the radar plot of the raw responses of sensors at different wavelengths for various chemicals. In addition to acetone, ethanol and isopropanol, coffee, cola and orange juice were tested. The ethanol and isopropanol samples were diluted using the same method used for acetone, while the cola and orange juice samples were pure. The coffee sample was prepared by dissolving 1 g of instant-coffee powder in 4 mL of water. All samples were heated and injected using the previously described method for the acetone sample. Some of the chosen sensor wavelengths were based on the known absorption wavelength of the single chemicals, while those for complex chemical were set to cover the full range of wavelengths, since they are not associated with a specific absorption frequency.

**Figure 14:**
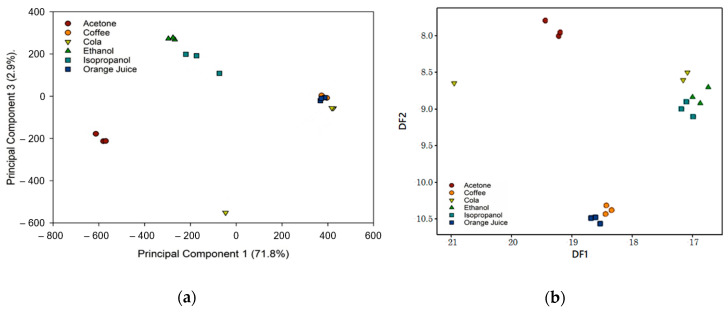
Classification analysis for testing of chemical standards and complex mixtures: (**a**) principal component analysis; and (**b**) linear discriminant analysis (DF—Discriminant Function).

Most applications of E-noses use classification analysis to distinguish between two or more groups and ultimately attempt to build and train a model that can be used as a discriminatory tool (e.g., ripe vs. unripe fruit in food quality control). Common classification techniques for E-nose datasets include principal component analysis (PCA) and linear discriminant analysis (LDA). PCA is an unsupervised linear method, which reduces the dimensionality (e.g., number of features) of the data, by selecting a small number of linearly uncorrelated principal components (PC) that explain the majority of the variation in the data [44]. LDA is a supervised linear method that tries to find a linear discriminant function to separate between datasets [45]. The commercial software package MultiSense Analyzer (JLM Innovation, Tübingen, Germany) was used to conduct the PCA and LDA analysis, as shown in Figure 14. Feature extraction was completed using the “maximum value” approach. This value can be easily calculated by subtracting the baseline from the maximum sensor response, for a given sample (previously referred to as: differential voltage sensor response).

The results in Figure 14 demonstrate that, for both analyses, distinct sample clusters with little to no overlap were created. This indicates that there is good separation between the sample groups. Isopropanol and ethanol are from the same class of chemicals and are therefore close to each other in both sets of analysis. In the future, these experiments could be repeated using at least three analyte concentrations to verify the device performance (regarding analyte discrimination), independent of the concentration.

## 4. Discussion

Unlike classical gas sensor technologies, which are based on transduction principles (e.g., electrical resistance, potential and current, frequency), optical sensors measure changes in light properties. The key advantage of this method is that it is able to overcome some of the limitations associated with typical MOX and CP sensor-based E-noses. To the best of our knowledge, the developed optical E-nose instrument is the first device that operates based on FPI sensors with tuneable filters. This technology is known to be less susceptible to influences from environmental parameters, such as humidity and temperature. However, further gas rig testing is required to characterise these effects on the sensors deployed in the E-nose. Other sensor parameters, such as sensitivity, detection limits, repeatability, response and recovery times, are also worth further investigating. Due to the operating principle of the optical E-nose, the response and recovery times are very rapid (less than a second). Therefore, the response time is that of the system and of the sample rate, not of the sensor. Since the addition/removal of the sample is controlled using the valve-pump negative-pressure system, the effects of altering the pump flow rate could be investigated.

Traditional E-noses use an array of sensors to analyse a chemically complex sample. In our system, not only do we have an array of four optical sensors, but these sensors can scan across a range of frequencies. In use, we can use specific frequencies (or a range of frequencies) to behave as “virtual” sensors to form arrays of different sizes. Furthermore, different combinations of these virtual sensors can be used for specific applications, making the instrument more flexible than a traditional E-nose.

The unit could also be used as a traditional gas analyser, but it would be necessary to determine the detection limits for a number of potentially relevant compounds, such as CO_2_, CH_4_, ozone, carbon monoxide, oxygen, ammonia and sulphur dioxide [46]. However, this is not necessarily needed for E-nose applications, since these focus on analysing complex mixtures. An alternative use is to use this E-nose to produce a so-called total VOC (TVOC) reading. The concept of TVOC attempts to report the combined effect of different VOCs, which may not be otherwise captured, due to low concentration levels of some contributing VOCs [47]. In this case, the E-nose would be set to scan the widest possible range of wavelengths and use these measurements to calculate a single value reading for air quality. To the best of our knowledge, an optical sensor has not been used to establish TVOC readings.

In the case of traditional quality assurance and quality control (QA-QC) applications, E-noses are likely to be in a controlled or possibly even laboratory environment to assure product uniformity in manufacturing is maintained. However, E-noses are being increasingly required to operate in uncontrolled conditions, such as open-air for environmental monitoring [48] and patient wards for point-of-care testing [49]. Food quality control protocols are also moving towards “spot-checks” which are conducted during transit or upon delivery of good [50]. For these applications, the sensor array must have low sensitivity to a wide variety of environmental parameters, but in particular to temperature and humidity [17]. In these situations, the benefits of utilising optical gas sensing technology is likely to be greatest. However, these environments also bring rise to new challenges.

There are some other limitations and drawbacks associated with the developed unit. In its current implementation, the sample vials need to be connected to the device inlet manually for analysis. This is both time-consuming and vulnerable to human error. An automatic injection system could be added to address this drawback. There are commercially available autosamplers, suitable for sample headspace analysis, which include agitator functionalities and allow the method parameters to be configured. In addition to these modifications, there may be some opportunities to further miniaturise the sensor configuration by implementing a more compact gas chamber design [51]. This could reduce the overall dimensions of the system and allow the internal components to be housed in a more portable enclosure.

Further scope for improvement relates to the transmission of sample data. The system currently connects to a laptop or PC via wired USB connection. In order for the device to be more practical in an outdoor or clinical setting, and keep up with current technological trends, the optical E-nose could be fitted with a Wi-Fi and/or Bluetooth compatible microcontroller to interface with a smartphone app or transmit data directly to a cloud-based computing platform for data analysis [52]. A previous publication has demonstrated how E-nose devices can be designed or upgraded to be Internet-of-things (IoT) enabled [53]. The appropriate data protection measures would also have to be implemented in order to ensure secure transmission and storage of data. Lastly, further work is also needed to ensure the environmental tolerance of the unit, in harsh conditions, and the addition of data processing capabilities to remove background signals.

## 5. Conclusions

In this paper, we show the development and testing of a novel optical-based E-nose, with an array of NDIR detectors. The optical E-nose is comprised of four emitter–detector pairs, which are encapsulated in individual heated sensor chambers. The inlet is controlled using a valve and a pump is used to create a negative-pressure system. Real-time data is presented to the user on a laptop. The functionality of the system was demonstrated in three sets of experiments. The first involved the testing of individual gases (CO_2_ and CH_4_), at different concentrations. The sensor responses showed that the response is linear to polynomial (second degree) for changes in CO_2_ and CH_4_ for the tested concentration range. Moreover, the results from this test indicate that the sensitivity for CO_2_ is below 25 ppm, and below 1 ppm for CH_4_. The second set of experiments involved testing chemical standards, as well as some complex mixtures and attempting to discriminate between these. PCA and LDA analysis demonstrated that the developed system was able to distinguish between the odour fingerprints of acetone, ethanol, isopropanol, coffee, cola and orange juice. The developed system has shown potential to serve as a discriminating tool for individual chemicals and groups. Further work will focus on determining the detection limits for different compounds and evaluating how this novel technology performs in different E-nose applications, including air quality monitoring and the analysis of food and biological samples.

## Figures and Tables

**Table 1:** List of sensors used in the optical electronic nose.

Manufacturer	Type	Optical Wavelength
InfraTec	LFP-3144C-337	3.1–4.4 μm
InfraTec	LFP-3850C-337	3.8–5.0 μm
InfraTec	LFP-5580C-337	5.5–8.0 μm
InfraTec	LFP-80105C-337	8.0–10.5 μm

**Table 2:** Differential voltage sensor response to 1000 ppm carbon dioxide.

Gas Chamber Length	Differential Voltage Sensor Response
10 cm	55.4 mV
20 cm	77.9 mV
30 cm	103.3 mV
40 cm	58.0 mV
